# A qualitative study exploring experiences of treatment in paediatric rheumatology - children’s, young people’s, parents’ and carers’ perspectives

**DOI:** 10.1186/s12969-025-01063-w

**Published:** 2025-01-23

**Authors:** Kassie Gracella Putri, Sunil Sampath, Charlotte Lucy Richardson, Alice McCloskey, Adam Pattison Rathbone

**Affiliations:** 1 School of Pharmacy, Faculty of Medical Sciences, Newcastle University, King George VI Building, Queen Victoria Road, Newcastle-upon-Tyne, NE2 4RU UK; 2 Great North Children’s Hospital, Newcastle upon Tyne NHS Foundation Trust, Newcastle-upon-Tyne, UK; 3 Faculty of Science, Liverpool John Moore’s University, Liverpool, UK

**Keywords:** Qualitative research, Treatment adherence, Patient lived experiences, Medicines use, Children and young people, Carer experiences, Behavioural science

## Abstract

**Background:**

There is limited literature in paediatric rheumatology describing holistic lived experiences of medical treatment from perspectives of children and young people (CYP) and their parents or carers (PC). This is important as it could have implications for adherence. This study aimed to explore treatment experiences of CYP and PC in a paediatric rheumatology service.

**Methods:**

Participants were recruited at a day-case unit for intravenous infusions at a tertiary paediatric rheumatology centre. Joint qualitative semi-structured interviews with CYP and PC were used to collect data. Data were transcribed, quality checked and thematically analysed using NVivo 12.4 to identify findings.

**Results:**

Thirty-two participants (15 CYP between the ages of 6 and 16 years, 17 PC) took part in interviews lasting 41 min and 43 s, on average. Participants described experiences using infliximab, followed by tocilizumab and abatacept. Participants experienced a wave, oscillating between positive and negative trajectories. Experiences of medical treatments were described as temporary, eventually changing and leading to treatment changes or cessation. Behaviours were influenced through somatic factors (pain, function), social factors (advice from health professionals, encouragement from friends, family and teachers, practicality of using treatment in relation to school, work and finance) and cognitive factors (fear of needles, fear of specific medications, beliefs about necessity).

**Conclusions:**

Collectively, findings demonstrate experiences of medical treatment reflect the nature of many paediatric rheumatology conditions, oscillating between periods of positive and negative trajectories. Somatic, social and cognitive experiences can be positive, when treatment is considered ‘successful’. Negative somatic, social or cognitive experiences led to behaviours such as treatment non-adherence. A limitation of the study is interviews were conducted jointly with CYP and PC, which may have influenced what participants were willing to say in front of one another however this does mean findings relate to both CYP and PC and so could be suitable targets for interventions to improve adherence.

## Background

Despite being relatively rare, several inflammatory rheumatic diseases are more prevalent in the UK than others. Juvenile Idiopathic Arthritis (JIA) has an age-standardised incidence and prevalence of 5.61 and 43.5 per 100,000 individuals respectively [1]. The incidence of Juvenile-onset Systemic Lupus Erythematosus (jSLE) is between 0.36 and 0.46/100,000 [2]. Juvenile dermatomyositis (JDM) is a rare muscle disorder leading to weakness and skin rashes and affects 3 children/1,000,000 annually [3, 4]. This evidence shows paediatric rheumatologic diseases, although rare when considered as individual conditions, collectively represent a significant population of children and young people (CYP, defined as from birth to 25 years old) [5]. Although there are pathophysiological differences between rheumatic diseases, the somatic, social and cognitive experiences of the diagnostic pathways and treatment services are similar [6]. Treatment here relates to medical or pharmacological interventions, but in practice can also include rehabilitative, occupational and psychological interventions. Recent work in the Lancet, identified psychological or cognitive issues were a research priority for patients with jSLE, JDM and JIA, as well as the health professionals looking after them [7]. There is an opportunity then to consider the experiences of a population of children and young people, by examining experiences of paediatric rheumatology services across disease states, rather than through a disease-specific approach.

Many of the conditions have similar symptoms and treatments, yet, studies investigating experiences are predominately focused on one condition, symptom, or medication [8–10]. Though there may be benefits to considering experiences of treatment across disease states, there are notable differences between some diseases. For example, families with children with JIA spend on average $1,686 more than other families on medication bills, more regularly visit doctors, and have more diagnostic tests [8]. Additional work has shown the social and economic cost (such as impact on employment and educational absences, as well as travel to and from appointments, in addition to the cost of the actual medication) is high for both CYP and their parents and carers (PC) [11]. The number of joints affected by a disease is also associated with higher treatment costs, implying a worse disease state may lead to more experiences of economic hardship [12]. Although this does suggest differences in experiences across rheumatic diseases, inflammatory diseases do share a similar pattern of disease progression, involving a series of relapses and remissions over time [13]. However, there is limited recent research exploring experiences of treatment across different diseases in paediatric rheumatology [10].

Furthering knowledge and understanding about experiences of CYP and PC in paediatric rheumatology across disease states is important as this could enable interventions to be developed which are applicable across the population accessing paediatric rheumatology services. Interventions to improve adherence are known to be effective [14], however the effect size can be reduced when deployed to wider populations [15]. This may be because interventions are typically developed using data from specific sub-groups, for example a specific disease, symptom or treatment, rather than collectively across populations and across disease states. Understanding experiences of treatment across populations and disease states in paediatric rheumatology, may be beneficial, as interventions may then retain their effect size when used across paediatric rheumatology services, increasing cost effectiveness.

Interventions to improve adherence in paediatric rheumatology are needed as injectable therapies, which are the majority of treatments prescribed for these conditions, are associated with lower adherence - although newer oral biologics, such as Tofacitinib, are becoming popular [16, 17]. Although evidence in adults suggests an openness to the use of injectable therapies, with some preference for intravenous (IV) over subcutaneous (SC) routes [18, 19], other studies have reported injectable medications used in endocrinology and immunology are associated with higher treatment anxiety, for both CYP and PC [20–22]. Further evidence generated during the pandemic suggests adherence to injectable therapy is lower for those with longer disease duration and less systemic involvement [23]. This evidence may have implications in paediatric rheumatology, were treatment experiences of anxiety and distress could lead to behaviours which reduce treatment adherence or lead to treatment cessation. However, there is limited work exploring experiences of using injectable treatment in paediatric rheumatology settings, which includes the perspectives collectively of CYP and PC, which could enable targets for interventions to improve adherence to be developed. Further work is therefore needed to describe experiences of treatment collectively, for children, young people, parents and carers.

Interventions to improve treatment experiences are typically based on cognitive and social psychology. In adults, treatment adherence is explained using the common-sense model of self-regulation (CSM), which describes health behaviours [24, 25]. The model assumes people actively and continuously go through problem-solving when faced with a health issue using ‘common sense’. For example, patients are given a stimulus which represents illness, referred to as a ‘health threat’ or ‘illness representation’ (e.g., a symptom, a blood test reading or other information). Experiences of the health threat can lead to the performance of coping behaviours to manage it. This could include behaviours such as visiting a health professional, attending an appointment or using treatment as prescribed. These behaviours are then appraised, using a combination of prior personal knowledge and current symptoms as well as social, cognitive and cultural experiences. Following appraisal, these behaviours (or ‘coping procedures’) can be repeated if they successfully managed the health threat, adapted if they partially managed the health threat or stopped if they made no difference to the health threat [24, 25]. However, this model was developed using evidence from adults’ perspectives and must be applied with caution to CYP or their PC.

Adherence to injectable treatments may represent a selection of coping behaviours, to manage the stimulus of symptoms, such as pain and loss of function. In a multi-centre study that explored barriers to treatment adherence, pain related to injectable and infusion therapies was a commonly reported by CYP ( [26]) However, CYP and PC may appraise injectable treatment with experiences of distress or anxiety, consequently adapting their coping behaviours, to reduce the health threat, by not attending appointments for treatment or withdrawing from services. This is further complicated as applying theory developed for adults to CYP may be problematic, as the latter population is still cognitively developing. Evidence suggests as children grow through adolescence and into adulthood, the responsibility for treatment adherence increases, with the perspectives and practicalities of using medicines, attending appointments and consenting to treatment subject to change [15]. Existing evidence suggests interventions to improve adherence for CYP specifically can be successful despite developmental changes they experiences [14]. However, this evidence was not specific to paediatric rheumatology or injectable therapies and did not consider PC perspectives, so may have limited application in practice.

One disease-specific study about JIA reported PCs felt their children’s childhoods were ‘stolen’ and CYPs with JIA felt different to other children without JIA [27, 28]. More recent work has focused on psychological characteristics of PC (such as impulsiveness and aggression) as well as other characteristics (such as the number of children being cared for) as important factors relating to treatment experiences [29]. This evidence artificially grouped participants into ‘good adherence’ and ‘bad adherence’, which may not reflect the dynamic experiences of everyday life (for example, having good adherence one week and bad adherence the next) [29]. This is further complicated as there are multiple models of understanding treatment adherence which are typically based on patient (rather than parental or carer) perspectives. Neither the CSM, the biopsychosocial model (which posits adherence is an experience linked to physical or somatic experiences as well as cognitive and social factors), and the Necessity Concerns Framework (which dichotomises adherence as a predictable response to beliefs about the need for, or concerns about side effects, of using a medication) accommodate PC perspectives [30]. More recent work has explored adherence as a social phenomenon, whereby social norms constructed through experiences of interacting with medicines, healthcare professionals and society influence medication use behaviours [31, 32]. However, this too did not include experiences or perspectives of PC. Treatment adherence in paediatric rheumatology are likely to be behaviourally, socially and cognitively demanding for PCs (as well as CYP), as they navigate new, complex systems whilst also meeting the developmental needs of a CYP they care for [33]. Further evidence of the modern, everyday lived experiences of injectable treatment are needed which consider both CYP and PCs perspectives.

Aims.

The aim of this study was therefore to explore children’s, young people’s, parents’ and carers’ experiences of treatment in paediatric rheumatology services.

## Methods

### Aim, design and setting

In-depth semi-structured qualitative interviews with CYP accompanied by PC were conducted in a paediatric rheumatology service at the Great North Children’s Hospital (GNCH) [34]. A topic guide, based on themes identified through reviewing published academic literature was used to guide the interviews conducted between March 2023 and June 2023. Participants provided informed consent (if over 8 years old) or assent to take part in the study (if under 8 years old). Participants did not receive any payment, compensation or any other inducement to take part. Ethical approval for the study was given by the Faculty of Medical Sciences Research Ethics Committee (Reference number 33823/2023) and the project was registered on the Trust’s Clinical Effectiveness Register.

### Research process

A convenience sample was recruited by one author (SS) who identified candidates for participation during routine clinical work. Candidates were given information about the project aims and processes. If candidates consented/assented, they were introduced to the other authors (KGP, APR) at their next routine hospital appointment. Two authors (KGP, APR) provided additional information, provided an opportunity to ask questions, assessed capacity to take part in research and took consent (or assent, if applicable). Interviews were conducted by one author (KGP), supervised by another (APR), with both the child or young person, alongside their parent or carer. Interviews were conducted in a private cubicle whilst the child or young person waited for/received parenteral treatment on a day-case ward. Interviews were recorded, transcribed, quality checked, anonymised and then audio recordings were deleted to protect confidentiality. Transcriptions were quality checked by reading through the transcript whilst listening to the audio recording to identify errors, which were subsequently amended by agreement of two authors (KGP, APR). Transcripts were anonymised by removing or replacing identifiable information such as names, places and other identifying characteristics. Participants were not contacted to verify the transcripts. Participants were recruited until theoretical data saturation (TDS) was reached (this is the point at which no further information was identified during interviews) [35]. TDS was identified through consensus by all authors. No age specific developmental methods were used to encourage children to participate in the interviews. Although this method was suitable for collecting data, the data may have been influenced by the age and developmental stage of CYP and the presence of their PCs during the interview.

### Analysis

Analysis was completed by one author (KGP) under the supervision of the other authors (SS, APR) using a method previously used [31]. Supervision entailed weekly meetings to review the analysis, interrogate coding and create consensus. Thematic analysis included (1) familiarization with the data by re-reading transcripts line by line, (2) ascribing primary codes to data by summarizing it in a word or phrase to identify structural (what happened) and textual (how it happened) findings, (3) secondary inductive coding included clarifying meaning through comparison to other codes within the data set and using imaginative variation to consider meaning and links between codes, (4) clustering codes together to identify common ideas, factors, and findings, (5) transforming clusters into relevant and understandable themes, by combining clusters, reflecting on our own biases and comparing the themes to data to ensure meanings were not lost [35, 36, 37, 38]. Coding, clustering and thematic grouping were discussed at regular supervision meetings where analysis was interrogated to identify similarities and differences between codes to promote methodological rigour [35, 39]. Supervision meetings included at least three authors, drawing on expertise of qualitative social science, health research and clinical expertise. NVivo Version 12.4 was used to maintain an audit trail during analysis which was also reviewed during supervision meetings to improve credibility. Analysis was audited by two authors (CLR, AM) who reviewed the findings, the analysis file and the full data set.

## Results

### Participant characteristics

Data saturation was reached at 32 participants, consisting of 15 CYP and 17 PCs (see Table 1). CYP were between ages of 6 to 16 years, with an average age of 12 years. The average duration of the interview was 41 min and 43 s with a range from 22 min 4 s to 1 h, 17 min. Juvenile Idiopathic Arthritis (JIA) was the most frequent diagnosis, with uveitis and joint damage being the most common comorbidity. The most common treatments the participants were prescribed were infliximab, followed by tocilizumab and abatacept (see Table 2). Duration of treatment varied between a year and 10 years, with an average of 5.5 years.

**Table 1:** Summary of Demographic Data

Characteristics	Total *n* (%)
Children	15 (46.9)
Parents	17 (53.1)
**Sex**	
Male	11 (34.4)
Female	21 (65.6)
**Age**	
Children’s Age, mean (range)	12 years old (6–16)
**Diagnosis**	
Juvenile Idiopathic Arthritis	12 (80)
Juvenile Dermatomyositis	1 (6.7)
Chronic Recurrent Multifocal Osteomyelitis	1 (6.7)
Linear Scleroderma	1 (6.7)
**Comorbidities**	**18**
Biomechanical musculoskeletal pain	3 (6.7)
Cataract	1 (6.7)
Chronic Fatigue	1 (6.7)
Glaucoma	1 (6.7)
Hypermobility	1 (6.7)
Joint damage associated with JIA	4 (26.7)
Lower limb length discrepancy	1 (6.7)
Obesity	1 (6.7)
Osteochondral defect associated with JIA	1 (6.7)
Uveitis	4 (26.7)

**Table 2:** Participants experiences of treatments in paediatric rheumatology

**Current Treatment Plans** ***n*** **(%)**	
**Infusions**	
Infliximab	5 (33.3)
Pamidronate	1 (6.7)
Tocilizumab	4 (26.)
Abatacept	4 (26.7)
Systemic Steroids	5 (33.3)
Rituximab	1 (6.7)
**Tablets**	
Mycophenolate Mofetil	4 (26.7)
Azathioprine	1 (6.7)
Hydroxychloroquine	1 (6.7)
**Injections**	
Methotrexate	2 (13.3)
**Eyedrops**	
Dorzolamide/Timolol	1 (6.7)
**Past Treatments**	
**Infusions**	
Abatacept	1 (6.7)
Baricitinib	2 (13.3)
Infliximab	2 (13.3)
IVIG	2 (13.3)
Tocilizumab	3 (20)
**Injections**	
Adalimumab	9 (60)
Etanercept	1 (6.7)
Methotrexate	11 (73.3)
Intra-articular Steroid Injections	11 (73.3)
**Tablets**	
Mycophenolate Mofetil	1 (6.7)
Sulfasalazine	3 (20)
**Eyedrops**	
Steroid Eye Drops	3 (20)

### Themes

Findings demonstrated experiences of treatment in paediatric rheumatology appeared to oscillate between positive and negative trajectories mediated by somatic, social and cognitive factors. Somatic factors related to biological, physical, functional experiences such as feeling pain, joint swelling, or limited mobility. Social factors related to social commitments and public aspects of everyday life, such as missing school, attending work and relationships with other people, such as healthcare professionals or family and friends. Finally, cognitive factors represented psychological beliefs or responses described by participants. These factors appeared in participants’ experiences of diagnosis, treatment initiation and adherence. Thematic findings are grouped below into Somatic, Social and Cognitive factors applying to both CYP and PCs. Each theme is described in detail below and exemplar data extracts to add further detail which are representative of the data are shown in Table 3.

**Table 3:** Data extracts

Theme	Diagnostic Experiences	Treatment Experiences
Oscillation	“[We were] very confused [during the diagnostic process]. Because we were, so going down this avenue of rheumatic fever and everything [the diagnosis] was just changing all the time […], So I was feeling very overwhelmed and confused by it sometimes.”– P23, Mum of child with Juvenile Idiopathic Arthritis “It was scary [at the beginning], we’re that used to it that there’s not a fear of it anymore, really, because we know what we’re dealing with, but, obviously, my thought is how it’s going to impact her in later life. How it’s going to affect her going forward for jobs, and you know that, how she’s going to cope with certain situations, yeah.”– P29, Mum of Child with Juvenile Idiopathic Arthritis “And even now that I know, like what it is and what’s happening, it’s still very scary to see, like, what’s going to happen to me like in the future?”– P12, Child with Juvenile Idiopathic Arthritis “Yeah, I think you kind of got a bit used to it. And you know now that if she has an illness [flaring], you probably know you’ve got to [go to the] hospital and she’ll be in flares. She just -- It is still scary, but not how it was before. You get used to it; you do. Yeah.”– P20, Mum of child with Juvenile Idiopathic Arthritis	“It starts, looks good for a while. Stops, looks too bad. Start a new one, yeah, it goes all around the circle, starts again and again and again.”– P1, Child with Juvenile Idiopathic Arthritis “It’s just never ending, so it’s, you’ll get used to a drug, and then we’ll get put into a different one. It’ll work for a little while and then it stops working, which just doesn’t ever seem to keep anything under control? It’s just very up and down all the time. Highs, lows, and it’s just -- it just feels like it’s never ending.”– P20, Mum of child with Juvenile Idiopathic Arthritis “Do I think she’ll probably change medications again? Yes, I don’t think she’ll stay on this for very long. I think she’ll end up coming off it again soon and going on to something else. [.] So, I think it’s just– nothing definite, and that’s how I kind of feel.”– P20, Mum of child with Juvenile Idiopathic Arthritis “She didn’t tolerate it well at all. She was sick all the time and stuff. As soon as we come into the rheumatology team and told them, and asked what was happening [to her], they changed her drug.”– P31, Mum of child with Juvenile Idiopathic Arthritis
Somatic Factors	“Yeah, I felt like very scared because I went from like being able to, like, run around and play and then not being able to do anything.”– P12, Child with Juvenile Idiopathic Arthritis “Yeah, Well, it’s just a condition, really, isn’t it? It’s -- I don’t know how to explain it. Felt a bit bad, having arthritis. But at the same time, it’s a condition that could be *controlled*, from what I’ve read about it and now knowing bits and pieces about it, *so it’s not life threatening*.”– P27, Dad of child with Juvenile Idiopathic Arthritis “Yeah, he does miss some school from joint pain. He restricted, like playing out, for the length of time he can play football, or the length of time we can go out for a walk, you know, he has to take multiple rests. I think he knows he’s not the same as every other 14 year old lad, but there is worse out there. Do you know what I mean? So he still gets up, gives it a go, and then the next day, that’s when it hits him [the pain]. So, the next day he’ll be sore, or he’ll be tired from whatever we did the day before.”– P18, Mum of child with Juvenile Idiopathic Arthritis	“I think it has been effective, it does work for times and she’ll be fine. And at the moment, touch wood, her legs are fine because sometimes she’s unable to walk, she’s unable to get out of bed, and she can’t even walk up and down the stairs. *The medication does help and it does keep it controlled to a certain leve*l.”– P20, Mum of child with Juvenile Idiopathic Arthritis. *“It makes her well*,* just like any other normal [kids]*. Yeah, she’ll run round. She’ll play.”– P25, Mum of child with Juvenile Idiopathic Arthritis He doesn’t feel like it, really, it [the treatment] doesn’t do anything. I suppose it does [work], cause they’re saying that sometimes there’s no active arthritis, so you know [it should work]. […] I suppose it probably does, but from Johnny’s perspective, he’s probably not [feeling that it’s effective]. He doesn’t really feel like much difference really.”– P18, Mum of child with Juvenile Idiopathic Arthritis “She’s *never really had any [side] effects* from it. it’s always been quite straightforward, which has been *good*, really.”– P16, Mum of child with Juvenile Idiopathic Arthritis “The side effects after the infusions were awful. I’ve never seen him that poorly like that in his life. That was *really worrying* though.”– P22, Mum of child with Juvenile Dermatomyositis “That made her upset, she was in a lot of pain with it, which obviously then made her frightened to have it. She was starting to hyperventilate because it was starting to hurt her. So, when that happened, it was like, ‘this isn’t working’ now, [because] she’s starting to really panic every time she’s having that [medication], *obviously we need another solution*.”– P29, Mum of child with Juvenile Idiopathic Arthritis “*It’s horrendous*. […] She gets quite a lot of illnesses because of the immune suppressants. She had impetigo about 3 weeks ago and that’s spread all over her face, so she needed antibiotics for that. *So it does have a massive effect on her school*,* and she gets tired a lot as well*.”– P20, Mum of child with Juvenile Idiopathic Arthritis
Social Factors	“[He’s] still doing his day-to-day thing, jumping on the trampoline, going out with his friends.”– P27, Dad of child with Juvenile Idiopathic Arthritis “Yeah, just followed what the doctor said. I mean, we didn’t know too much about the condition to start off with.”– P28, Mum of child with Linear Scleroderma	*Pro-treatment* “Because the nurses told me that I’d be damaging him in a long term if [he] didn’t have it, that was what they said.”– P18, Mum of child with Juvenile Idiopathic Arthritis. “He’s a big believer of what’s the point in coming. But I’m -- I don’t know. I’m kind of one of those people where I do what I’m told to do. And if the doctors told me to do something, then we have to listen -- *and we have to do it because they’re doctors*.”– P18, Mum of child with Juvenile Idiopathic Arthritis “No, don’t think I am [worried about the side effects]. It *doesn’t affect that much day-to-day life*, and she still managed to do everything that she wants to do. It’s not really an issue.” -- P28, Mum of child with Linear Scleroderma “They always let me change the hours [of my shift] when I need to get her, like the other week she was downstairs [the rheumatology clinic] [.] They stopped your treatment, and then we had to come back on that [medication] a couple of days later, but I was supposed to be at work that day, so we had to ring [my] work and say, ‘I’m not coming in, can I swap it [my shift], I’m going to come in another day,’ and they just say ‘that’s fine.’ ”– P28, Mum of child with Linear Scleroderma “We just take the letters in from the hospital and try to explain why [she was absent], I mean, *we picked that school because they already had a child in there that has JIA*.”–P31, Mum of child with Juvenile Idiopathic Arthritis. “I think, yeah, you definitely are looked after here. It’s a really good hospital to be at, and you just know that they know what they’re talking about.”– P16, Mum of child with Juvenile Idiopathic Arthritis “But we do have good family support, so if it’s not me, it’s his mum or your grandma or your sister or, you know, somebody in the family. So, we manage.”– P21, Dad of child with Juvenile Idiopathic Arthritis “Our work has been really *supportive*. They give us the time off to come in and bring Isla [to the hospital], so that’s not an issue.”– P25, Mum of child with Juvenile Idiopathic Arthritis *Anti-treatment* “School, we’ve had big issues with school just because of his absence, so he’s home schooled now actually. They were OK initially, weren’t they, the school? But then I think as you got older, they just didn’t [understand], Because you obviously had quite a lot of absences […]initially, they were fine for him to come in late if he was struggling, but then it just became more difficult and difficult [for school to be fine with it], and so as he grows older, I think they just expected him to just be ready to go every day and attend school. So now he’s now being tutored at home.”– P21, Dad of child with Juvenile Idiopathic Arthritis. “Money’s been a bit of a worry, especially mainly on travel. That’s the only thing really, I wouldn’t not come up here. I know she needs it, but it’s the money that [creates] worry the most.”– P31, Mum of child with Juvenile Idiopathic Arthritis “So, it’s a big chunk out of that sort of thing, that was one of the reasons why I don’t work. We didn’t know how often she would have to come to the hospital, whether she’s having to go for physio, or where she’s having to go for hydrotherapy. So, [it] takes time out of my day, *whereas if I was working*,* I wouldn’t be able to do that*, because it would impact on my work life.”– P29, Mum of child with Juvenile Idiopathic Arthritis “Even at school, and like I say, some of the nurses didn’t believe me, so I had to take photographic evidence of my own child being sick, which is not very nice. *It literally took about two years to fight to get him off that medication*,.”– P18, Mum of child with Juvenile Idiopathic Arthritis “It was quite difficult because, like I say, I didn’t drive. His dad was always working, and his siblings were babies. So, it was quite hard.”– P18, Mum of child with Juvenile Idiopathic Arthritis
Cognitive Factors	“It’s scary. When you’re looking into it and what it is, it’s just wondering how it is going to affect [her physically in] the long term…”– P31, Mum of Child with Juvenile Idiopathic Arthritis “[The diagnostic process] was long because we didn’t know what was happening. And I think, if we’d had an idea of what it was, then we could have understood the process. But initially, we didn’t know anything about it, and she was gradually getting worse, so we were totally in the dark right at the beginning.”– P29, Mum of child with Juvenile Idiopathic Arthritis “There’s a certain amount of relief, I think, when you find out what it [the disease] is.”– P24, Dad of child with Juvenile Idiopathic Arthritis	“[We went to the psychologist] because of her methotrexate. The injections were really bad, and she was really struggling with the blood tests, and they suggested putting her into the psychologist here and we started to see her. We saw Charissa [Psychologist’s Pseudonym] for a little while, didn’t we? And she just, talked to her, kind of try to get to the root of the problem and try to understand what it was. And she has got a lot better since we saw her.”– P20, Mum of child with Juvenile Idiopathic Arthritis “I think the methotrexate was the worst one. I hated that every week. It was terrible. I don’t know [why], it was just a mental block because, like, I just couldn’t press the button [for the self-injections] […] It’s not that it hurts. It was just -- the feeling I just like, just knowing what was happening.”– P7, Child with Juvenile Dermatomyositis “Like the second time, I ended up screaming and crying cause I hated it so much because I developed a fear of needles.”– P2, Child with Chronic Recurrent Multifocal Osteomyelitis I think I just didn’t like it because of the needles, really.”– P14, Child with Juvenile Idiopathic Arthritis “When we went to psychologist– and to be fair, they were fairly honest straight away, they said ‘we’ve seen the injections, we really don’t know how we’re going to get her to like them, but we’ll do what we can to like, try and give her choices.’ […] and she [Isla] actually just told me that she didn’t feel like herself anymore and she didn’t want to be here anymore. So that’s when rheumatology [team] got involved and said, ‘Right. OK, then let’s change everything [treatment regimen].”–P25, Mum of child with Juvenile Idiopathic Arthritis

### Somatic factors

Somatic factors described participants’ physical or biological experiences and were linked to both diagnosis and treatment. For example, participants reported manifestations of rheumatological disease led to somatic symptoms of pain and reduced mobility which intersected with their interactions with the physical world (such as being able to use apparatus in playgrounds). These symptoms were also used to appraise if treatment was successful, where if a treatment improved symptoms enabling engagement with the physical world it was considered successful, a positive trajectory for treatment adherence behaviours. However, the return of symptoms or experience of side effects (such as vomiting) were perceived as treatment failure, contributed to a negative trajectory. If appraisal showed a negative trajectory, participants reported experiences of changing treatment adherence behaviours, representing a turning point back to an upward or positive trajectory of adherence behaviours, whereby somatic experiences would improve (i.e. symptoms or side effects would not interfere with interactions with the physical world like being able to play in the playground). Experiences of symptoms of pain and reduced mobility may have a biological, physiological cause, but the findings here describe a relationship between adherence and the physical world. For example, reduced mobility meant one participant could not be physically active enough to play football, interacting with the physical world by kicking the ball, running up and down the pitch, etc. It was not necessarily the biologically-caused, physiological-response of reduced mobility that was problematic, but rather the somatic or physical experience of not being able to interact with the physical world around him (such as, the ball, the pitch) in the way he desired. Similarly, one parent reported their experiences of fatigue from the additional physical demands of caring, such as lifting, carrying and moving making them too tired to do other things, or physically sitting in hospital waiting rooms causing discomfort after they’d left hospital. These findings demonstrate adherence behaviours may go beyond biological or physiological responses, but rather the somatic elements of interacting with the physical world.

### Social factors

Social factors were identified as experiences linked to social agents or actors, such as family, friends, social institutions (like schools or hospitals) and social norms (such as ‘going to work’ or ‘out with friends’). Although ‘being social’ did appear to be linked to diagnosis (and represented ‘wellness’), it was also used to appraise treatment adherence behaviours. These experiences oscillated between ‘pro-treatment’ trajectories and ‘anti-treatment’ trajectories. For example, participants reported being given information by healthcare professionals (such as doctors and nurses) in a child-friendly format made them feel cared for. Another reported being given time off work or and having good social support networks were ‘pro-treatment’ and support adherence behaviours. Participants also reported social factors which were linked with negative treatment appraisal, such as the cost of transport to the hospital, headteachers sending letters about school non-attendance linked to hospital appointments, missing social events with friends, and meeting demands of other family members, created a negative trajectory. This finding suggested every day social interactions with others about accessing treatment contributed to CYP and PC adherence behaviours.

### Cognitive factors

Cognitive factors were feelings, emotions or strongly held beliefs experienced as peaks of relief and troughs of fear. Cognitive experiences linked to diagnosis were linked to fear of the unknown, which changed to relief when a diagnosis was given. Fear was also identified in relation to treatment formulations (injectable) or specific medications (methotrexate) which changed to relief once a first dose had been administered or medications were changed. Fear was described as negative and reduced treatment adherence behaviours, though participants did report input from psychologists and counselling about treatment formulation and specific medications had a positive impact on treatment adherence. Both CYP and PC in this study had beliefs and thoughts which appeared to have an overarching inclination to ‘not-want-to-need’ injectable treatment but ‘accepted’ injectable treatment as ‘a necessary, but temporary evil’ for the time being. Both CYP and PC believed that the effectiveness of their injectable treatments would eventually peak and then slowly reduce, which would mean treatment would be stopped and changed to another medication.

## Discussion

What is pervasive in the data is an oscillation, whereby participants have negative somatic, social and cognitive experiences which reduced treatment adherence until a ‘turning point’ of positive somatic, social and cognitive experiences which supported treatment adherence. However, this trajectory ‘turned again’ negatively during treatment, leading to treatment non-adherence, such as switching treatments, stopping treatment or requiring further intervention with non-pharmacological support (such as psychological counselling). This oscillation between positive and negative trajectories appears to reflect the flare-remission-flare nature of rheumatological diseases more broadly. The findings also mirror existing models of understanding health behaviour; where somatic, cognitive and social factors reflect the biological, psychological and social domains of the biopsychosocial model for both CYP and PCs.

### Implications in context of existing research

Previous work has identified the importance of somatic and social experiences in paediatric rheumatology [40]. This also aligns to theory in the CSM, the biopsychosocial model, and the NCF, whereby somatic symptoms as well as social and cognitive factors, represent triggers, stimulus, beliefs about necessity or ‘health threats’, which prompt behaviours which facilitate treatment adherence, such as visiting a health professional, starting new treatments or continuing to engage with therapeutic interventions [25]. Additionally the findings echo work from 2012, which described similar experiences of oscillation in children and adolescents with JIA, between hope and despair [41] and work from 2016 describing an ‘emotional rollercoaster’ for children with JIA and their parents [42]. This is expected given the high number of participants in this sample with JIA. The findings presented may go beyond existing work by demonstrating somatic, social and cognitive experiences may be used to appraise treatment adherence behaviours by both CYP and PC across disease states in paediatric rheumatology, rather than just JIA. However there were limited numbers of participants with other rheumatic diseases and so further work is needed to verify this.

Like all qualitative research, although the study had a small number of participants, it adds to the literature as it considers the experiences of the paediatric rheumatology population in different disease states, rather than as individual conditions, and as a whole group, rather than specific groups of CYP or PCs. Collectively this population represent a sizable group who access similar treatments, diagnostic pathways and health services [2, 3, 43]. Understanding their experiences of treatment and what this brings to everyday life, how this influences motivation to perform adherence behaviours may enable targets for interventions to improve services to be identified at scale. For example, this study identified being able to meet social expectations, by ‘going to work’ or ‘out with friends’ were important social experiences which resulted in positive treatment appraisal and treatment adherence. Existing work has also identified the importance of understanding PC values when making decisions about treatment [8] however little is known about how social and cognitive factors influence prescribing decisions or how these inform consultations to stop, change or continue treatment for healthcare professionals. The findings presented by this study, indicate social experiences may be a factor influencing adherence as it contributes to positive or negative adherence trajectories and treatment appraisal. Previous studies have reported social factors which influence treatment adherence, though key parts of shared-decision making ideologies in healthcare, are missing from consultations with CYP and PCs [44]. This means policy makers and practitioners must consider how treatment influences social lives of CYPs and PC when designing treatment protocols, pathways and delivering services.

Interventions to improve treatment adherence behaviours using social and cognitive factors are reported in relation to other disease states [31, 45]. These interventions adopt a social constructivist theoretical approach, to consider the intersection of systems of healthcare, family, commerce, media and law which patients use to interpret and appraise their symptoms and treatment. This study takes these findings further, extending this approach to CYP and PCs. Identifying supports to enable CYP and PCs to continue to interact with the physical world and social life are needed, for example through improved availability of accessible recreational spaces, with adequate facilities to manage health needs (e.g., clean rest rooms, accessible waste bins) or through the prescription of physical therapy to support rehabilitation (e.g. reducing the impact of movements like running or kicking). In paediatric rheumatology, these could include integrating treatment times with social events for young people or carers or providing resources (like internet access, private spaces, work stations) for CYP or PCs to continue to work productively or engage with education whilst waiting for their appointment or receiving treatment. Alternative spaces to deliver treatments should also be considered, which could reduce social disruption when receiving treatment, though of course this must be tempered with a clear understanding of the complications, and risks, of delivering paediatric rheumatology services from a patient safety perspective [46]. Effective management of many paediatric rheumatic conditions requires a multidisciplinary team including physiotherapists and occupational therapists who are instrumental in aiding the CYP to regain their physical function. This can help CYP to integrate back into regular physical activities at home and school. Further work is needed to explore broad social action which may also be required to educate employers, headteachers and others, to react flexibly to absence requests and help with costs of treatment socially and economically [11], reducing the social burden CYP and PC face when trying to adhere to treatment.

### Limitations of the study

A limitation of the study is interviews were conducted jointly with CYP and PCs, which may have influenced what participants were willing to say in front of one another. Additionally, the data related to both CYP and PC collectively, and so there is little understanding of how the perspectives differed between the groups or where one group may have more intense experiences. Additionally, the cognitive age of CYP was not assessed prior to participation Furthermore, recruitment of participants via the clinical team may have meant participants felt biased towards the clinical team (i.e., recruiting favourable participants). This sampling bias does not appear to have influenced the findings as these were not directly about the service being received, but rather broader experiences of treatment. The sample was made up largely of CYPs diagnosed with JIA, with fewer participants with other diseases. Further work is therefore needed to verify findings across disease states. The work adopted standardised methods by using a topic guide, quality checking transcriptions, and reviewing the analytical audit trail which increases trustworthiness [39]. The methods used are reported transparently and in sufficient detail for the study to be reproduced, increasing the dependability and credibility of the findings [39]. The data was collected from a single site in North East England and the qualitative nature of the work means the findings are not generalisable however may be transferable to similar settings. Although the study identified factors which may influence treatment adherence in paediatric rheumatology, further work is needed to consider the depth and intensity of these experiences and how this relates to the development stage of CYP -although this is likely to vary from child to child and carer to carer.

## Conclusion

The aim of the study was to explore treatment experiences of children, young people (CYP) and their parents or carers (PC) in a paediatric rheumatology service. Although the work did not consider the intensity and depth of experiences, the findings describe positive and negative trajectories of treatment which oscillated between peaks of relief and troughs of fear. Somatic, social and cognitive experiences were used to appraise treatment success. Where treatment was considered successful from a somatic, social or cognitive perspective, further treatment adherence behaviours were performed (and vice versa if treatment considered unsuccessful). This echoes the biopsychosocial model of health, the NCFs and the CSM, demonstrating the intersectionality between the physical world, social norms and cognitive processes in relation to treatment adherence but contextualises these theories to CYP and PC in paediatric rheumatology. A key take away message is that although somatic factors, like the physical response to treatment are important, so too were the cognitive and social factors, like feeling cared for and being able to continue to meet social commitments, like attending school or going to work. The experience of positive and negative trajectories in treatment raises new questions about what ‘treatment success’ or ‘treatment failure’ means and how peaks or troughs can be identified and discussed in consultations with CYP and PCs. Finally, the experiences reported by this study suggest a multidisciplinary approach to medical treatment is needed, to address the biopsychosocial needs of this population.

## Data Availability

The dataset supporting the conclusions of this article is included within the article and its additional files.

## Declarations

### Author information

KGP was an MRes student working under the supervision of SS and APR. SS is a Paediatric Rheumatology Consultant in at the Great North Children’s Hospital, AM is a Reader in Pharmacy Practice at Liverpool John Moore’s Hospital, CLR is a Senior Lecturer in Pharmacy Practice at Newcastle University, and APR is a Lecturer in Clinical and Social Pharmacy at Newcastle University and Advanced Clinical Pharmacist at the Children’s Hospital.

## Abbreviations

JIA: Juvenile Idiopathic Arthritis
jSLE: Junenile-onset Systemic Lupus Erythematosus
JDM: Juvenile dermatomyositis
UK: United Kingdom
CYP: Children and Young People
PC: Parents and carers
